# Construction of individualised care programmes for patients with pancreatic cancer with postoperative weight-loss control based on the Delphi method: a cross-sectional study in China

**DOI:** 10.1186/s41043-024-00525-3

**Published:** 2024-03-01

**Authors:** Leying Chen, Zhongyan Huang, Qiuju Tian, Qinghua Zha, Shiyu Zhang, Zhe Chen, Ziyun Dong, Yuqing Zhou, Ming Zhang, Xiaoyan Wei

**Affiliations:** 1 Department of Pancreatic Surgery, Shanghai Ruijin Hospital Affiliated of Jiaotong University, No. 197 of Ruijin No. 2 Street, Huangpu District, Shanghai, 200025 China; 2 Department of Nurse Management, Shanghai Ruijin Hospital Affiliated of Jiaotong University, Shanghai, 200025 China

**Keywords:** Pancreatic cancer, Weight loss, Personalisation, Scheme construction, Delphi method

## Abstract

**Background:**

At present, clinical nutritional care for patients with pancreatic cancer focuses more on the observation of the effect of enteral parenteral nutrition, and there is a lack of personalised care plans for weight-loss control. We used the Delphi method to construct a set of personalised nursing programmes to effectively control the rate of postoperative weight loss in patients with pancreatic cancer.

**Methods:**

This study was a cross-sectional investigation. Through literature analysis, literature review and data review, a personalised nursing plan for the postoperative weight-loss control in patients with pancreatic cancer was preliminarily developed. From October to December 2022, the Delphi method was adopted to conduct two questionnaires for 32 experts working in fields related to pancreatic diseases in Grade-A tertiary hospitals from four different departments. After statistical processing, the personalised nursing plan was determined according to the perceived level of importance, coefficient of variation, full score rate and recognition rate of the indicators.

**Results:**

The recovery rates of the two rounds of consultation were 93.75% and 100%, respectively, and the overall authority coefficient of the experts was 0.918, which represented ‘authoritative’. In terms of importance, the coefficient of variation was 0–0.137; in terms of feasibility, the coefficient of variation ranged from 0.09 to 0.194. Finally, a scheme consisting of 36 entries in 8 dimensions was built. This programme is comprehensive in content, meets the nutritional diagnosis and treatment needs of patients in the stage of postoperative rehabilitation, provides relatively comprehensive nutritional assessment and support and has a robust system and feasibility.

**Conclusions:**

The individualised nursing plan for patients with pancreatic cancer with postoperative weight-loss control based on the Delphi method is highly scientific and reliable and has positive significance.

## Background

Pancreatic cancer is a tumour of the digestive system and has an insidious incidence, high malignancy and low 5-year survival rate. Recently, the results of a data analysis based on the Global Burden of Disease Study showed that the incidence rate of pancreatic cancer in China is not optimistic and is on the rise at an average rate of 2.04% per year [1]. Involuntary weight loss is one of the most significant clinical manifestations of pancreatic cancer, and 70–80% of patients with pancreatic cancer will experience varying degrees of weight loss [2], of which nearly one-third of patients show significant weight loss (weight-loss rate ≥ 10%) after surgery [3]. Studies have shown that a common challenge in the postoperative care of patients with pancreatic cancer is postoperative weight loss. The underlying causes of postoperative weight loss involve both pathologic and physiologic factors. Pathological factors include issues of tumour growth and aggressiveness, where the tumour may occupy space in the pancreas and interfere with normal pancreatic function and metabolism, which in turn can lead to diabetes mellitus and indigestion. Aggressive tumours may also exert pressure on surrounding organs, affecting the passage of food. Some tumours may also produce metabolites that further interfere with the patient’s metabolism. Physiologic factors include decreased appetite, absorption problems and metabolic changes that may result from the surgery itself, nausea, vomiting and decreased appetite that may result from chemotherapy and radiation. This is as well as pain and psychological problems that patients may experience. Understanding these factors is therefore crucial. Individualised care plans should take into account the patient’s specific pathology and physiology to provide interventions supported by scientific evidence to mitigate the effects of postoperative weight loss and improve quality of life. It is therefore important to provide personalised care plans for patients with pancreatic cancer when dealing with postoperative weight loss [4–6]. The relationship between the nutritional status of patients with pancreatic cancer and weight loss is extremely close [7], and at present, clinical nutritional care for patients with pancreatic cancer focuses more on the observation of the effect of enteral parenteral nutrition, etc. Oral nutritional intervention is reported as effective in increasing nutritional intake and improving the quality of life in patients with cancer who are malnourished [8]. However, contrary to this, there have been limited benefits from nutritional support reported in patients with cancer in receipt of chemotherapy, in the preoperative period or in those patients with advanced-stage disease [9, 10]. In addition, there are few studies on postoperative weight-loss control nursing for patients with pancreatic cancer, insufficient standardised nursing programmes and effect evaluation indicators and no unified consensus on the content and methods of postoperative weight-loss control nursing. The aim of this study is to establish a set of personalised nursing plans for patients with pancreatic cancer after radical surgery by using the Delphi method as the basis of a preliminary investigation to effectively control the rate of postoperative weight loss in patients with pancreatic cancer to reduce postoperative complications, control medical costs and improve the work efficiency of nurses. Finally, it will provide an invaluable reference for future research and clinical practice.

## Methods

### Establishment of the study group

The study group consisted of eight members, including one deputy chief nurse, three chief nurses and four nurse practitioners. The members included one specialist nutrition nurse and one specialist oncology nurse.

### Initial construction of a personalised care plan for postoperative weight-loss control in patients with pancreatic cancer

**Fig. 1:**
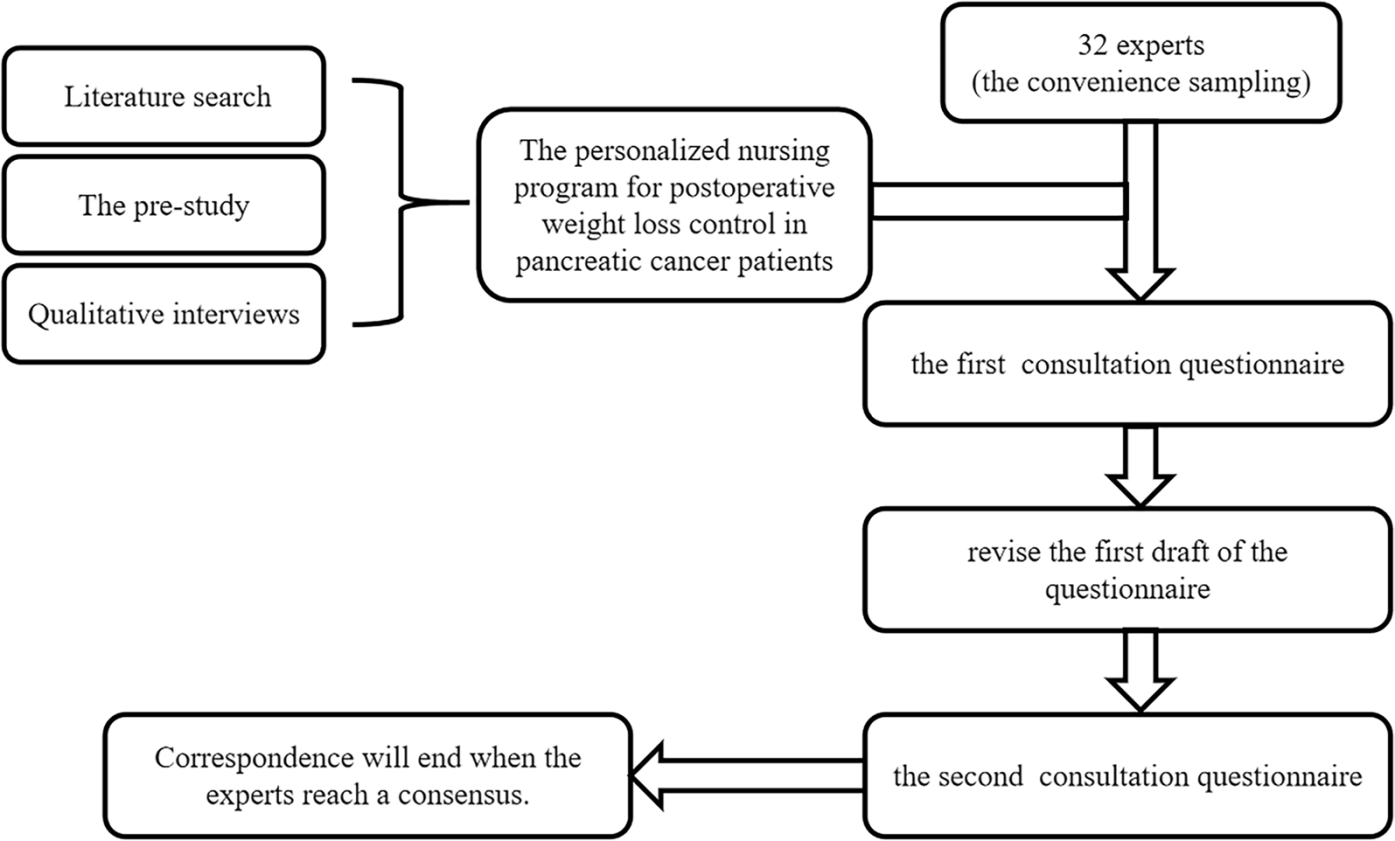
Study design process

This research mainly included preliminary research, literature search, the preparation of an expert consultation questionnaire and the selection of correspondence and telecommunications experts. The ratings and written comments of the experts on our study subjects were compiled, counted and analysed, and the final protocol was revised on this basis. See Fig. 1.

#### Literature search

The literature search of Chinese databases mainly included China Knowledge Network, Wan fang, China Biomedical Literature and Wipro Chinese Biomedical Journals; the English literature search mainly came from PUBMED, EMBASE, Medline, Cochrane Library and JBI. The English database was based on the MeSH subject terms ‘pancreatic neoplasms’, the subject terms ‘nutrition therapy’, ‘nutritional status’, ‘weight loss’ and the free terms ‘pancreatic cancer’, ‘tumours or cancer of the pancreas’, ‘carcinoma’, ‘pancreatic ductal’, ‘pancreatic intraductal neoplasms’, ‘diet therapy’, ‘nutritional support’, ‘physician-nurse cooperation’, ‘individualised’, etc. To avoid miss detection, the Chinese search terms were all free words, including ‘pancreatic cancer’, ‘pancreatic tumour’, ‘pancreaticoduodenectomy’, ‘weight-loss nutritional intervention’, ‘nutritional care’, ‘nutritional support’, ‘nutritional supplementation’, ‘diet’, ‘personalisation’, ‘individualised’, ‘multidisciplinary collaboration,’ etc. The timeframe for searching the literature was from the start date of the database up to the end of December 2022.

A total of 10 guidelines [11–20], 6 expert consensuses [10, 21–25] and 12 relevant secondary literature [26–37] were included. To ensure the quality of the included guidelines, they were analysed using the AGREE II evaluation system [38]; the JBI Centre for Evidence-Based Health Care’s Literature Quality Evaluation Tool (2016 version) was used to complete the systematic evaluation as well as to certify the quality of the expert consensuses [39]. The quality of the 10 guidelines and 6 expert consensuses was Grade A. Of the 12 systematic evaluations included, eight were of a high quality of evidence, two were of moderate quality, and the other two were of low quality of evidence.

#### Survey of the current status of perioperative weight loss in patients with pancreatic cancer and analysis of factors affecting it [40]

The aim of this study was to investigate patients who underwent radical surgery for pancreatic cancer between September 2021 and February 2022 at Ruijin Hospital, affiliated to Shanghai Jiaotong University. It also sought to understand the current status of perioperative weight loss in patients with pancreatic cancer and to analyse the underlying factors using generalised estimating equations. Eventually, 180 patients completed all the investigations from the time of admission (T0) to the stage of preparation for discharge (T3). For details, see reference 34.

#### Qualitative interviews with patients with pancreatic cancer and their primary caregivers

One-on-one, face-to-face, semi-structured interviews were conducted with the respondents to capture the real experiences, problems, needs and suggestions for weight loss and dietary and nutritional care that existed in the two groups of patients with pancreatic cancer and their primary caregivers. The interviews included 8 postoperative patients with pancreatic cancer and 8 primary caregivers corresponding to the patients, totalling 16 individuals. Within 24 h of completing the interviews, the relevant work was done, and the audio recordings and transcripts were converted into a unified text form. Next, the authenticity and completeness of the relevant documents were checked by two researchers.

### Delphi method of expert consultation

#### Preparation of expert consultation questionnaire

In the first expert consultation questionnaire, the following was covered. The questionnaire was divided into four parts: the first part detailed the background, objectives and importance of the study and requested the consent of the experts. Instructions for completing the form were provided so that the respondents could fill in the questionnaire correctly. In the second part, questions were set to find out detailed information about the experts: their work unit, length of service, position, specialty, education and title. The third part was the experts’ self-assessment form, which reflects their level of expertise in their field of study and the criteria for judging them. The fourth part is the experts’ rating of the importance and feasibility of each entry in the research programme. The experts evaluated the projects based on their judgment using a Likert 5-point scale. In terms of ‘importance’, the five options ‘not important, not too important, generally important, important and very important’ corresponded to the scores 1, 2, 3, 4 and 5, respectively. For ‘feasibility’, the five options of ‘not feasible, not too feasible, average, feasible and very feasible’ corresponded to scores of 1, 2, 3, 4 and 5, respectively. If the score was lower than three, the experts were required to provide comments regarding modification and deletion in the remarks column. If the experts had additional comments, they could make additions in the ‘Entries to be added’.

#### Selection of experts

Adopting the convenience sampling method, 32 experts in this field were selected from October to December 2022. The qualification criteria for the experts were as follows: 1. working in a tertiary-level hospital and having been engaged in pancreatic disease-related fields for more than 5 years; 2. having a bachelor's degree or above, and a title of intermediate or above; and 3. having the intention to participate in the study and having participated in two or more rounds of correspondence in the course of the study. The questionnaires were distributed in paper or electronic form to the participants, and they were returned after being completed independently. The research team revised the first draft of the questionnaire based on each expert's comments to create a second round of questionnaires. A summary of the first round of comments was attached to the second round of questionnaires and the correspondence was conducted in the same manner. Correspondence ended when the experts reached a consensus.

### Principles of programme revision

When assessing the importance and feasibility of dimensions and entries, if the mean value of the rating is < 4 and the coefficient of variation is > 0.25, the experts can make suggestions for additions, deletions or modifications. Before making the final decision, these suggestions needed to be discussed by the research team to determine the retention, deletion or modification of items in the programme.

### Ethics statement

This study has been reviewed and approved by the Ethics Committee of the hospital (2022 Pro-Lun Audit No. 249) and has passed the Evidence Summary Registration of the Evidence-Based Nursing Centre of Fudan University (No. ES20233043).

### Statistical analysis

Statistical analysis was performed using SPSS 25.0 software, setting the test level at *α* = 0.05. Basic information about the experts was analysed descriptively. The questionnaire recovery rate was used as an indicator to measure the degree of active participation of the experts in the study. The degree of authority of experts was measured by the coefficient of authority (Cr), which was calculated based on the mean values of the coefficient of basis of judgment (Ca) and the coefficient of familiarity (Cs) [41]; `*x* ± *S* standard deviation was used to assess the degree of centralisation of the experts' opinions, which reflected the degree of difference in the importance ratings of the entries. In contrast, the degree of harmonisation of the experts’ opinions was measured by the coefficient of variation (CV) and Kendall’s coefficient (*W*), reflecting the degree of agreement in their opinions [42].

## Results

### Background information of the participating experts

Thirty experts in fields related to the diagnosis, treatment and care of pancreatic diseases were included in this study. The basic information of the experts is shown in Table 1.

**Table 1:** Basic information on Delphi consultants

Project	Categorisation	Number of people	Component ratio (%)
Sex	Woman	12	40
	Man	18	60
Age	25–30	8	26.67
	31–35	11	36.67
	36–40	5	16.67
	41–45	3	10
	46–50	3	10
Years of experience	5–10	18	60
	11–19	7	23.33
	20–29	3	10
	30–39	2	6.67
Education	Undergraduate	6	20
	Bachelor's degree	3	10
	Doctoral degree	21	70
Title	Middle level	18	60
	Deputy Senior	10	33.33
	Regular senior	2	6.67
Working section	Pancreatic Surgery	22	73.33
	Pancreatic Oncology	3	10
	Clinical Nutrition	2	6.67
	Department of critical care medicine	3	10

### Degree of expert participation

In the first round of correspondence (the background and objectives of the study, the information about the experts, the experts’ self-assessment form and the importance and feasibility of each entry in the research programme), 32 questionnaires were distributed, and 30 of them were successfully returned with an effective recovery rate of 93.75%. In the second round of correspondence (a summary of the first round of comments), 30 questionnaires were distributed, and all questionnaires were successfully returned, with an effective recovery rate of 100%. In the first round of correspondence, 21 experts (70% of the total) suggested amendments and deletions; in the second round of correspondence, four experts (13.33% of the total) suggested amendments. This indicates a high level of active participation of experts in the study.

### Degree of expert authority

The degree of authority of experts can be assessed using Ca and Cs. Ca includes four dimensions, and the degree of familiarity includes five dimensions, and the values assigned to each dimension are as follows (Table 2).

**Table 2:** Assignment of values to the level of expert authority

Judgment basis		Large	Medium	Small	
Practical experience		0.4	0.3	0.2	
Theoretical analysis		0.3	0.2	0.1	
References to domestic and international sources		0.2	0.1	0.1	
Intuition		0.1	0.1	0.1	
Familiarity level	Very familiar	More familiar	General	Not familiar	Unfamiliar
Expert self-assessment	1.0	0.8	0.6	0.4	0.2

It is generally believed that experts have good authority when the degree of authority is Cr ≥ 0.7 [20]. In this study, the individual authority coefficient of the experts was 0.80–1.00, and the overall authority coefficient was 0.918, which indicates that they have high authority, and the obtained correspondence results have high reliability. The authority coefficient of the participating experts is shown in Table 3.

**Table 3:** Table of expert authority factors

Expert number	Ca	Cs	total	Cr	Cr'
1	1	0.8	1.8	0.9	0.918
2	0.8	0.8	1.6	0.8	
3	0.9	0.8	1.7	0.85	
4	0.8	1	1.8	0.9	
5	1	1	2	1	
6	1	1	2	1	
7	0.8	0.8	1.6	0.8	
8	0.9	0.8	1.7	0.85	
9	0.9	1	1.9	0.95	
10	0.8	0.8	1.6	0.8	
11	0.9	1	1.9	0.95	
12	0.9	1	1.9	0.95	
13	1	1	2	1	
14	0.8	1	1.8	0.9	
15	1	1	2	1	
16	1	1	2	1	
17	0.9	0.8	1.7	0.85	
18	1	1	2	1	
19	0.9	0.8	1.7	0.85	
20	1	1	2	1	
21	0.9	0.8	1.7	0.85	
22	1	1	2	1	
23	1	1	2	1	
24	1	1	2	1	
25	0.8	0.8	1.6	0.8	
26	1	1	2	1	
27	0.8	1	1.8	0.9	
28	1	0.8	1.8	0.9	
29	0.9	1	1.9	0.95	
30	0.8	0.8	1.6	0.8	

*Ca* coefficient of basis of judgment, *Cs* coefficient of familiarity, *Cr* coefficient of authority

### Degree of concentration and coordination of expert opinions

The mean number of the first round of correspondence is 4.367–4.933, the CV value of importance is 0.051–0.198, and the CV value of feasibility is 0.113–0.249; the mean number of the second round of correspondence is 4.533–5, the CV value of importance is between 0 and 0.137 and the CV value of feasibility is between 0.09 and 0.194. After two rounds of correspondence, the harmonisation coefficients of the experts’ opinions on the importance of the indicators were 0.271 and 0.312, and the harmonisation coefficients of the opinions on the feasibility of the indicators were 0.259 and 0.324, respectively. These results were statistically significant (*P* < 0.5). The experts’ views on the entries converged and the results are reliable and harmonised. The correspondence is closed. See Table 4 for details.

**Table 4:** CV, W and significance test of the second round of expert correspondence

Project	First round of correspondence				Second round of correspondence			
	CV	*W*	*χ*^2^	*P*	CV	*W*	*χ*^2^	*P*
Importance	0.051–0.198	0.271	219.659	< 0.01	0–0.137	0.312	480.13	< 0.01
Feasibility	0.113–0.249	0.259	210.49	< 0.01	0.09–0.194	0.324	244.007	< 0.01

*CV* coefficient of variation

### Personalised nursing plan for postoperative weight-loss control of patients with pancreatic cancer

In the first round of expert consultation, there were 6 items with an expert score of less than 3 points, and a total of 20 written opinions were provided by the experts. Six items were modified, and two additional items were added. The specific content is as follows: (1) Adding critical care physicians to the multidisciplinary team building of nutrition management in pancreatic surgery. (2) In the team building dimension, add ‘improving the staffing of nursing teams and cultivating nutrition or pancreatic specialised nurses’. (3) Add the entry ‘Weight-Loss Grading System’. (4) Add the entry ‘After the patient undergoes the first nutritional screening and evaluation after admission, they can start creating a “personalised nutritional diagnosis and treatment file for the patient”’. (5) Modify the ‘Introduction Calorimetry Method’ to ‘Use an H-B Formula Prediction Method for personalised measurement of patients’ actual energy consumption’. If within 7 days after surgery, the oral route cannot reach 50% of the required amount, ‘modify to 60%’. Change the phrase ‘start enteral nutrition within 24 h after surgery’ to ‘start enteral nutrition as soon as possible after surgery based on the patient's condition’. ‘If there is a gastric emptying disorder, and the condition does not improve after 7 days’ should be changed to ‘If there is a gastric emptying disorder, it should be treated promptly, and the condition does not improve after 3–5 days’.

**Fig. 2:**
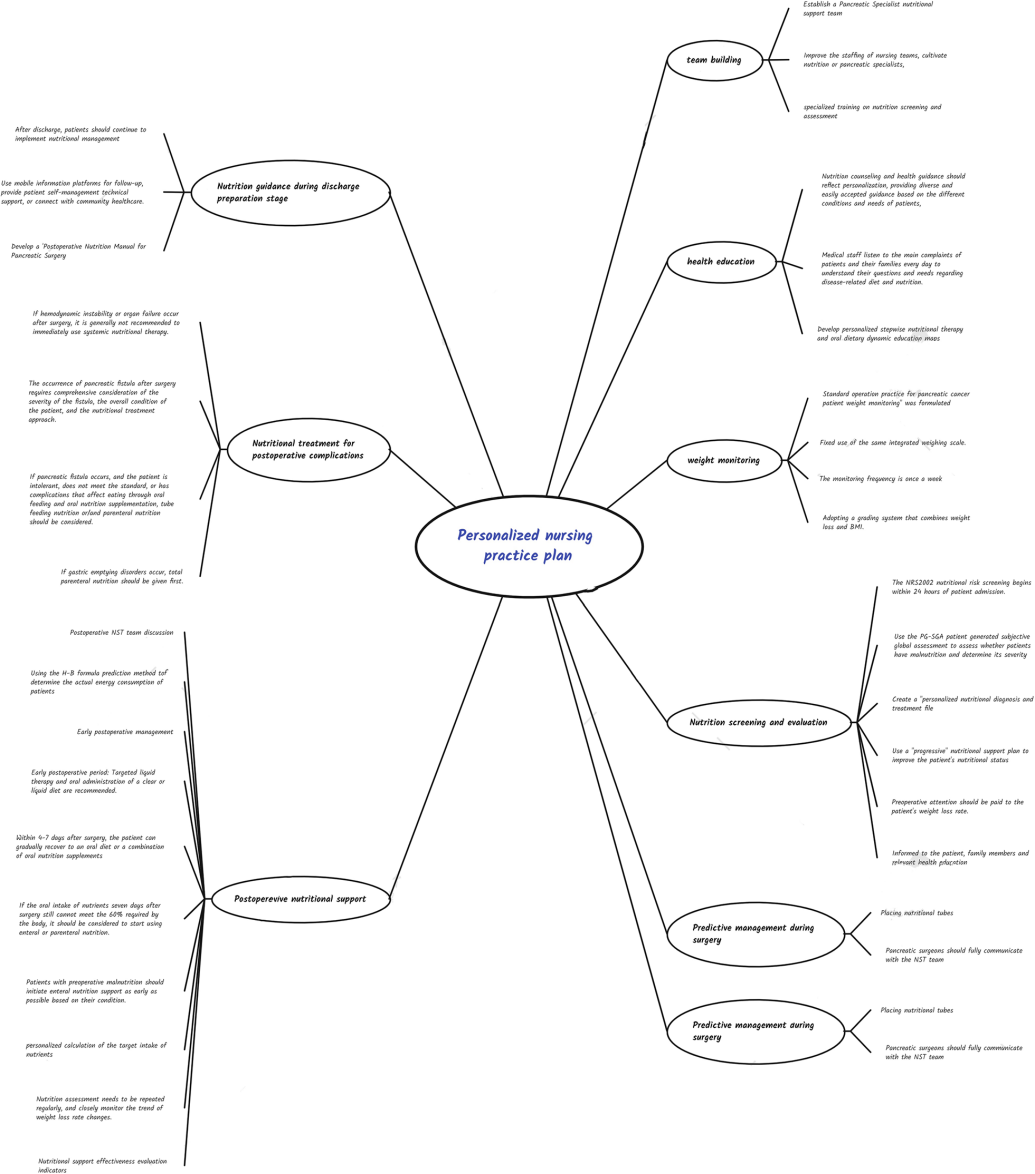
Personalised nursing practice plan for weight-loss control of pancreatic cancer patients

In the second round of expert consultation, the experts proposed four written opinions and modified three items. The experts wrote four opinions as follows: (1) The 15th expert gave a suggestion on question 9. ‘When a patient loses 5–10% of their weight, surgery should be performed as soon as possible with active nutritional support’. (2) The 14th expert gave an opinion on question 11, stating that ‘the surgical team must fully communicate with the ward nurses and nutritionists’. (3) The 14th expert provided an opinion on question 19, emphasising that the decision on which nutritional support plan to adopt should be based on the overall postoperative condition of the patient. (4) The 16th expert proposed a supplementary opinion, stating that ‘electronic nutrition assessment and patient nutrition records are recommended’. The specific content is as follows: (1) Add ‘Human body composition measurement when conditions permit’. (2) Add ‘Follow up using a mobile information platform after discharge’. (3) Add the ‘Food Caloric Equivalence Exchange Table’ to the ‘Postoperative Nutrition Manual for Pancreatic Surgery’. After two rounds of expert consultation, the personalised nursing plan for weight-loss control of patients with pancreatic cancer after surgery was finalised, including 8 modules and 36 items in total. See Fig. 2.

## Discussion

### The necessity of establishing a personalised nursing plan for weight-loss control in patients with pancreatic cancer after surgery

Recently, researchers have been paying increased attention to the impact of weight loss on the prognosis of patients with pancreatic cancer. This study found that patients with severe weight loss were relatively less responsive to treatment, had shorter survival and a significantly reduced quality of life [43–45]. Hashimoto et al. [46] pointed out that different degrees of postoperative weight loss in patients with pancreatic cancer were significantly correlated with the patients’ overall survival. In addition to the special pathophysiological changes caused by pancreatic malignant tumours, pancreatic surgical treatment can also affect the metabolic and nutritional status of the body to varying degrees, resulting in impaired internal and external secretion functions of the pancreas. The likelihood of body weight loss and malnutrition are significantly increased, which affects postoperative recovery and clinical outcome [23]. A retrospective analysis showed that the average weight loss of patients undergoing radical surgery for pancreatic cancer was 7.01% compared with that before surgery [47], and postoperative body mass index (BMI) decreased by an average of 1.75 compared with preoperative BMI [48]. It can be seen that the weight-loss rate of pancreatic cancer after radical surgery was significantly higher than that of oesophageal cancer [49] (3.71–5.13%), gastric cancer [50] (4.7%) and rectal cancer [51] (2.41%). Weight loss has a significant impact on the nutritional score, and greater weight loss means a higher score and a worse nutritional status, so it is especially important to maintain a balance of energy intake throughout the treatment process to prevent significant weight loss [52]. Evidence increasingly indicates that preoperative exercise and nutritional optimisation improve perioperative physical function in major abdominal surgery [53–55]. However, a recent scoping review of nutrition within rehabilitation oncology research identified that nutrition assessment was inconsistently applied [56]. Of the 110 studies reviewed, 37 (34%) included a nutrition treatment component. Only half of these studies provided the goal for the nutrition component of their rehabilitation programme and, of these goals, less than half referenced accepted nutrition guidelines [57]. Furthermore, a failure to diagnose and address malnutrition and dietary complications has been identified as the primary unmet supportive care need of patients with pancreatic cancer. Therefore, the measurement, determination and evaluation of weight loss is an indispensable part of the nutritional status assessment of patients. However, in the current clinical nutritional diagnosis and treatment of patients with pancreatic cancer, different medical centres and individual pancreatic surgeons select varying strategies, methods and timing of nutritional support to match different conditions after surgery. Therefore, it is necessary to build a more personalised clinical nursing practice to provide an important basis for clinical workers to control postoperative weight loss in pancreatic cancer patients.

### Scientific and reliable research results

The research team in this study invited front-line clinical experts, care managers, educators and nutrition experts in the field of pancreatic disease care to serve as correspondence experts. According to the results of this study, the effective recovery rate of the first round of correspondence questionnaire was 93.75%, and the effective recovery rate of the second round of correspondence questionnaire was 100%, indicating that experts were very active in participating in the research. The authority coefficient of the overall experts in this study is 0.918, indicating that the authority of experts in this field has reached a high level. In all the items of the second round of correspondence, the coefficient of variation was less than 0.25, and the W coefficient was higher than that of the first round, indicating that the opinions of experts showed a high level of consistency.

### Revelations of an individualised nursing plan for weight-loss control in patients with pancreatic cancer after surgery

The average importance of Pancreatic Specialist nutritional support team (PSNST) team building ranked first among all the items, indicating that the construction and collaborative operation of multidisciplinary teams in nutrition management is the basis of this nursing practice plan. A study by Shen Mingyan et al. [58] also showed that giving a full role to nurses in the team can significantly improve the postoperative recovery speed of patients with pancreatic cancer and maximise the prognosis of patients. The National Nursing Career Development Plan (2021–2025) clearly states that nurses play a vital role in the team. They are not only responsible for the day-to-day care of patients, but also key participants in the postoperative recovery process. Nurses are important members of the medical team and need to work closely with other members such as doctors, physiotherapists and social workers to develop and implement rehabilitation plans. Through the effective allocation of medical resources, a detailed preoperative assessment of each patient can be performed, including the patient’s physical condition, the severity of the condition and other factors that may affect the outcome of the surgery. This requires a precise diagnosis by a doctor and a thorough assessment by a medical team. On this basis, a personalised surgery and rehabilitation plan can be developed for the patient [59]. Finally, establishing an effective community medical cooperation network can also help patients recover quickly after surgery. We need to establish effective communication and cooperation with patient caregivers and families to ensure that they understand and cooperate with the implementation of treatment management programmes. At the same time, support and education are also needed to help them better participate in the patient's care process. Improving the professionalism and competence of nursing staff while improving the quality of patient care will result in greater benefits for our healthcare system and better care for patients [60]. The average importance of nutritional guidance at the stage of a patient’s discharge preparation ranked second. In the previous qualitative study, it was found that almost all subjects had a strong demand for dietary guidance at home after discharge, including what kind of food should be eaten after returning home, the amount of food, how to eat less and more meals and how to match different meals. All patients hope to receive detailed professional guidance, which is similar to the research results of Green et al. [61]. Jingjing Mou et al. [62] found that more than half of the respondents felt helpless at home and needed professional medical staff to assist them. By implementing discharge preparation services, we can achieve a tripartite ‘win–win’ situation for patients, hospitals and society [21].

### Advantages of personalised care for weight-loss control in patients with pancreatic cancer after surgery

In the previous literature quality evaluation of this study, it was found that although nutrition management of patients with pancreatic cancer has attracted more and more attention from experts in the industry, systematic reviews, evidence summary, expert consensuses and programme formulations have been carried out on this topic, but the diagnosis and treatment opinions of clinical patients often do not match the recommendations of the guidelines. The Chinese Society of Parenteral and Enteral Nutrition and the Pancreatic Surgery Group of the Chinese Society of Surgery jointly launched an exclusive survey of domestic experts. A snapshot survey of perioperative nutrition management was conducted among 96 physicians in 64 first-class hospitals in 35 cities in 22 provinces in China. The results showed that irregular and unreasonable nutritional support was widespread [63]. In addition, nutritional nursing strategies for patients with pancreatic cancer mostly focus on the content and methods of health education. Among the nutritional assessment indicators, from the perspective of patient self-management, body weight and BMI are the most intuitive, simple and accessible indicators. However, there is currently a lack of pancreato-related nursing programmes with weight loss as the observation index. In view of the differences of nutritional diagnosis and treatment guidelines for patients with pancreatic cancer at home and abroad, a more personalised clinical nursing plan for weight-loss control should be explored in line with China’s national conditions.

### Limitations

The implementation timeframe of this programme was only during the postoperative rehabilitation of patients with pancreatic cancer in the hospital, which has certain limitations, and it needs to be more widely applied in clinical practice to continuously verify its actual clinical guidance effect, promote the continuous improvement of this programme and lay a foundation for follow-up home-based self-weight management and nutritional management of patients.

## Conclusions

In this study, through qualitative interviews and subject extraction based on the actual needs of both parties, a personalised nursing plan for postoperative weight-loss control of patients with pancreatic cancer was constructed. The content of this programme is comprehensive, which can meet the needs of nutritional diagnosis and treatment of patients in the stage of postoperative rehabilitation, provide comprehensive nutritional assessment and nutritional support and has a good system and high feasibility.

## Data Availability

All data generated or analysed during this study are included in this published article.
